# How to mend a broken heart: a major stab wound of the left ventricle

**DOI:** 10.1186/1749-7922-7-17

**Published:** 2012-05-28

**Authors:** Mari-Liis Kaljusto, Theis Tønnessen

**Affiliations:** 1 Department of Cardiothoracic Surgery, Oslo University Hospital, Ullevål PB 4956 Nydalen, and University of Oslo, Oslo, 0424, Norway

## Abstract

A 28-year-old male admitted with a stab wound under his left nipple, underwent emergency surgery because of confusion, a decreasing blood pressure and increasing tachycardia. A median sternotomy incision was made and after establishing cardiopulmonary bypass, a 7 cm wound in the left ventricle and a smaller wound in the left atrium were repaired. An injured segment of lung was resected and the left anterior descending and circumflex arteries were grafted after weaning from cardiopulmonary bypass was initially unsuccessful. Although the patient suffered a stroke, probably due to prehospital hypoperfusion, he eventually recovered without major sequelae. In addition to the case report we present a literature review of the last 15 years pertaining the management of penetrating cardiac injury.

## Background

Onehundred and sixteen years ago Alex Cappelen repaired a penetrating injury of the left ventricle through a left anterior thoracotomy in Christiania (former name of Oslo), in one of the world`s least violent countries [1]. Cappelen`s operation is considered to be the first report of a cardiac surgical procedure. Today trauma centers all over the world perform complex cardiac repairs due to penetrating trauma but the mortality is still high [2-5].

We report the case of a young man who suffered a large stab wound (SW) in the left ventricle and left atrium in addition to a lung injury for approximately 2 h before undergoing reparative surgery. In addition we present a literature review of penetrating cardiac injuries from 1997 – 2012 (Table 1). As data source we used all available English-language articles from peer-reviewed journals in the Ovid MEDLINE and PubMed databases. The articles selected were relevant case reports, original articles and reviews focusing on the clinical presentation of penetrating cardiac injury, initial management, operative technique, complications and follow up.

**Table 1:** Overview of the papers on penetrating cardiac injury from 1997 to 2012

**Ref nr, author, year, journal and study origin. Study type**	**Patients/patient group/injury site**	**Outcomes/performed surgery**	**Key results**	**Comments**
[2] Asensio et al. (1998), J Trauma, USA. Prospective evaluation	2-year prospective evaluation of 105 penetrating cardiac injuries	65% GSW (survival 16%), 35% SW (survival 65%). EDT in 76 pts with 10 survivors (16%)	Presence of cardiac tamponade and the anatomical site did not predict outcome, presence of sinus rythm when the pericardium was opened did	
[6] Baker et al. (1998), Arch Surg, USA. Retrospective study + review	106 pts with penetrating heart injury (1989–1995): 60 GSW, 46 SW, 55% overall survival.	6 patients on CPB (4 gunshots, 2 stabs, only 2 GSW survived)	Few survivors due to long time from injury to CPB. Those who were resuscitated >5 min prehospitally had a very poor outcome. SR at admission- good prognostic sign. CPB no good to reverse outbled situation/profound shock, but necessary to repair multichamber injuries/large injuries	
[7] Bar et al. (2009), Ind J Thorac Cardiovasc Surg, Israel. Retrospective study	14 pts with penetrating cardiac wound requiring operation (1999–2006) (9 SW, 2 GSW and 2 schrapnel injuries, 1 multipl trauma)	4 sternotomies, 10 anterolat thoracotomies (8 with sternum transsection). 5LV, 6RV, 3RA injuries - all single chamber injuries, no combined.	No CPB, 100% survival, all discharged	Mean interval from injury to surgery 37 min
[8] Barbosa et al. (2011), Interact Cardiovasc Thorac Surg, Argentina. Case report	18 yr male, SW in 4th ic space in the left midclavicular line	Left thoracotomy, suture of right ventricular wound at admittance	Developed pneumonia/lung edema postop, after 30 days AVR for penetrated aortic valve and closure of shunt (RV -> aorta)	
[9] Bowley et al. (2002), Ann Thorac Surg, South Africa. Case report	24 yr male, multiple stab wounds	No vital signs, PEA, at EDT: tamponade. 2 cm LV wound with LAD transsection, transsected PDA on the opposite side (RV)	Initially Foley catheter in the wound, mattress sutures, LAD ligation, PDA ligation. VF, hypotension: OPCAB with right gastroepiploic artery . Died of respiratory complications due to Brown-Sèquard lesion (another stab injury to the spinal cord)	
[10] Burack et al. (2007), Ann Thorac Surg, USA. Retrospective study	207 pts with mediastinal penetrating trauma 1997–2003, 72 (35%) unstable.	72 unstabel pts, 15% had cardiac injury with 18% survival when explored in ED and 71% when reached OR	With penetrating mediastinal trauma the mortality is 85% when moribund at arrival and 55% when unstable (overall data, not injury specific)	
[11] Carr et al. (2011), J Trauma, USA. Retrospective study	2000-2009 penetrating cardiac injuries, both GSW and SW	28 SW with 17 survivors (61%), no information about anatomical site	Functional outcome (5yrs) after: if coronary arteries were not involved - good chance to normal cardiac function at follow up.	
[12] Chughtai et al. (2002), Can J Surg, Canada. Review + case report	Cases of 9 pts, 8 managed with CPB in trauma setting from 1992-1998	Only 2 pts of the presented had a sole cardiac injury (LV + coronary artery, RA + intrapericardial vena cava)	The patient with LV and coronary artey injury died (no CPB), the other patient survived without sequele	
[3] Clarke et al. (2011), J Thorac Cardiovasc Surg, South Africa. Retrospective study	All patients with penetrating cardiac injury requiring operation from 2006-2009	Of 1062 stab wounds, 104 were operated, 76 had cardiac injury, overall mortality 10%. Approx 50% median sternotomy, 50% left thoracotomy	When data put together with mortuary data: mortality of 30% for SW (in the mortuary cohort of 548 patients with SW, 38% had penetrating cardiac injury). Less than 25% with penetrating cardiac injury reach hospital alive, of these ca 90% survive.	Mostly SW, also mortuary data analyzed. The center has no availability for CPB.
[13] Claassen et al. (2007), J Trauma, USA. Case report	2 male pts : 21 yr and 27 yr	Pas 1: SW in 5th right ic space (axilla) (+ in abdomen), 400ml on chest tube + knife blade in thorax: laceration of right ventricular outflow tract (sutured) + lung resection	Pas 2: SW in left supraclav midline. Tamponade at FAST: pericardial drainage, thereafter stable. Sternotomy after transfer, laceration of the pulmonary outflow tract, sutured, further repaire of aortopulmonary shunt (thrill + TEE)	Think outside the box: SW outside the precordium
[14] Comoglio et al. (2010), Int J Emerg Med, Italy. Case report	75 yr male with chest pain and syncope, had been working with a nailgun	Stable, underwent CT where the nailgun nail was found imbedded in the left ventricular wall. Removed through median sternotomy, suture without CPB	The pt underwent formal coronary angiography to rule out underlying coronary disease	
[15] Desai et al. (2008), J Thorac Cardiovasc Surg, Canada. Brief communication	22 yr male, single SW in the left chest	Severe shock, loss of vital signs in the ED. EDT, ROSC after opening of pericardium. Tamponde + through-and-through laceration of the RV, stapled and transferred to OR	CPB, staples had occluded the PDA, the wound in close proximity. Staples removed, wound sutured. Intraoperative fluorescence coronary angiography showed widely patent PDA	
[16] Fedalen et al. (2001), J Trauma, USA. Case report	30 yr male, isolated SW to left anterior chest wall	Tension pneumothorax, hypotension, cardiac tamponade. Transfer to OR	Median sternotomy, proximal laceration of LAD with posterior wall of the vessel intact. OPCAB with SVG, intraluminal shunt. Laceration used as anastomotic site. Discharge at postop day 8	
[17] Fulton et al. (1997), Ann Thorac Surg, South Africa. Case report	61 yr male, a single SW in right 2nd ic space parasternally. History of right-sided empyema 18 yrs ago treated by thoracotomy and decortication	Stable, enlargened mediastinum at chest X-ray. Arcography showed laceration to innominate artery, left common carotid artery and left subclavian artery. Distal cannulation, repair in deep hypothermic arrest	Uneventful postoperatively, discharge at day 10	
[18] Hibino et al. (2003), Journal of Cardiac Surgery, Japan. Case report	39 yr male, SW anterior chest wall, suicide attempt.	Median sternotomy at OR. Injury of the right ventricular outflow tract, repair without CPB	2 yr after aorto-right ventricular fistula (dyspnea), repair with patch and AVR. The authors suggest long term follow-up to detect unindentified lesions	
[19] Ito et al. (2009), Gen Thorac Cardiovasc Surg, Japan. Case report	51 yr male, SW in left 5th ic space with the ice pick still in place, suicidal attempt	Ice pick was moving synchronously with heart beat, echo showed tip in right ventricle, cardiac tamponade	CPB, mattress stich. Heart murmur day 12, 5mm ventricular septal defect detected. No surgery, follow up	
[20] Jodati et al. (2011), Interact Cardiovasc Thorac Surg, Iran. Case report	24 yr construction worker, shortness of breath and palpitations, unaware of the pneumatic nailgun injury	Nail through RV outflow tract, interventricular septum, through the mitral valve at TEE and CT.	Median sternotomy, CPB. Entry point on RV, nail tip barely visible, not exit wound after LA was opened. Nail removed, anterior leaflet of mitral valve repaired. Discharge at postop day 5	
[21] Kang et al. (2009), Injury, New Zealand/Canada. Review	Review about causes of penetrating cardiac injury, pathophysiology, sequelae, initial and operative management	Hihglighted key points for every section, outlining of prognostic factors	Few other conditions in medicine are as lethal; death occurs from cardiac tamponade or exsanguination; the greatest danger is missing the dgn; resuscitation is of limited value; immediate operative intervention is the only meaningful treatment	
[22] Karin et al. (2001), Eur J Emerg Med, Israel. Case report and literature review	1. 29 yr male with single SW in left chest. 2. 35 yr male, stabbed in left lower thorax	1. Cardiac tamponade, ED thoracotomy: SW in the LV transsecting LAD (ligated, sutured). CPB with SVG in OR 2. Hemopneumothorax, respiratory distress, chest tubes. FAST: tamponade. Left thoracotmy at OR, distal LAD transsection, ligated.	Both had normal echocardiographies postoperatively and were discharged respectively 10th and 7th postop day	
[23] Kurimoto et al. (2007), Surgery today, Japan. Case report	57 yr male, SW in 5th ic space parasternally, suicide attempt	Arrest prehospitally, EDT at admission + pericardiotomy, further percutaneous CPB + repair at ED. 3 cm left ventricular wound near coronary artery	Postop encephalopathy, 3 yrs afterwards at rehabilitation home	
[24] Lau et al. (2008), Singapore Med J. Case report	31 yr male, 2 SW: in the left 4th ic space and in the right 2nd ic space	Pulseless with PEA, EDT, SW in the RV, internal cardiac massage to ROSC, transfer to the OR. Suture of the laceration	Discharged to further rehabilitation due to hypoxic encephalopathy	
[4] Molina et al. (2008), Interact Cardiovasc Thorac Surg, USA. Retrospective study	237 pts (2000–2006) with EDT for penetrating injury, of these 94 with penetrating cardiac injury	GSW 87%, SW 13%, overall survival 8% (5% for GSW, 33% for SW)	None of the patients who reached OR needed CPB. Predictors of survival: sinus rythm, signs of life at ED, SW vs GSW, transport by police, higher GCS	Mostly GSW -very poor outcome
[25] Moore et al. (2007), Am Surg, USA. Case report	16 yr male, multiple stab wounds	Tachycardia and hypotension, left hemothorax. FAST: pericardial and infraabdominal fluid. LAD injury (ligation), RV (suture).	OPCAB (SVG) due to evolving large anteroseptal MI. Abdominal packing. Discharge postop day 17.	
[26] Nwiloh et al. (2010), Ann Thorac Surg, USA/Nigeria. Case report	11 yr boy, arrow in the 4th ic space	Pt admitted 3 days after hunting with arrow in the midline. Attempted retracted at local hospital, referred to the visiting cardiothoracic team from USA. TTE: arrow through right ventricle, ventricular septal shunt	CPB, retraction of the arrow and suture of the RV. Shunt was insignificant, not repaired	
[27] O’Connor et al. (2009), J R Army Med Corps, USA. Review	History, demographics and outcome, repair techniques, special occasions etc.			Refer to iv adenosin infusion for temporary arrest to facilitate the repair
[28] Parra et al. (2010), J Thorac Cardiovasc Surg, USA. Case report	81 yr male struck by a stingray in his left chest	CT: left pneumothorax, foreign body through mediastinum. Left anterior thoracotomy at the OR, the barb was found imbedded in the heart, the entry was repaired and pt transferred to a cardiac center	At cardiac center: CPB, barb through both right and left ventricles. RA was accessed and the barb pulled out in an antegrade fashion. Ventricular septal and RV defects closed with pledgeted sutures. Discharge 60 days postop	Splenectomy due to hemorrhage postop day 1 (unidentified injury, the pt fell when attacked by the sting ray)
[29] Seamon et al. (2009), J Trauma, USA. Retrospective study	283 pts with cardiac or great vessel penetrating injury requiring EDT (2000–2007)	88% GSW (survival 2,8%), 12% SW (survival 24,2%)	Predictors of survival in multivariate analysis: GSW and GCS	Multiple GSW almost unsalvagable
[30] Sugiyama et al. (2011), Ann Thorac Surg, USA. Case report	20 yr male, SW in left chest (nipple level)	Cardiac arrest at ED, left anterior thoracotomy, suture of right ventricle	Postop instable, 7. day - 1,9 cm septal defect with left to right shunt (3,7-1), ARDS etc., shunt=VSD repaired 2 mnths afterwards	
[5] Tang et al. (2011), Arch Surg, USA. Retrospective study	406 pts with penetrating cardiac injury from 2000-2010	74% SW, 26% GSW. Overall survival 27%.	Focusses on postdischarge complications, 17% had an abnormal echocardiogram at follow-up; all managed conservatively	
[31] Tasdemir et al. (2011), Acta Cardiol, Turkey. Case report	19 yr male, SW left chest	Presented in shock, tamponade andcomplete bilat visual loss. SW of LV with LAD injury,	CPB, SV graft to LAD, visus gradually regained	
[32] Toda et al. (2007), Interact Cardiovasc Thor Surg, Japan. Case report	50 yr male, 3 SW by 30 cm sashimi knife, (Neck, 4th ic space, right upper quadrant of abdomen), suicidal attempt	Hypotensive, FAST negative, CT showed pneumopericardium and left hemothorax	Median sternotomy, RV laceration, repair by pledgeted sutures. LV laceration near posterolateral branch of CX, without bleeding, covered with TachoComb.	
[33] Topal et al. (2010), J Trauma, Turkey. Retrospective study	Penetrating cardiac injury (57 SW, 4 GSW), 2002-2009	53 left thoracotomies, 4 median sternotomies. 2 LAD injuries, ligated. Total mortality 15% (isolated RV −11%, isolated LV 31% (mixed SW and GSW).	95% injury in 1 chamber. Focusses on predictors of outcome: > mortality when uncouncious, BP<50, low Hct, Na, temp and PH. Patients pronounced “dead on arrival” were not assessed in this study.	
[34] Topaloglu et al. (2006), Tex Heart Inst J, Turkey. Case report	19 yr male, SW with skrewdriver in 5th left ic space	Dyspnea and hypotension, 1500ml chest tube output. Left anterior thoracotomy at OR, RV wound repair.	1 week later a cardiac murmur occurred, transfer to a cardiac center, TTE: perforation of membranous septum and anterior leaflet of the mitral valve. Median sternotomy, CPB, LA access: pericardial patchrepair of the leaflet, suture of the septal defect through RA. Discharged postop day 5.	
[35] Topcuoglu et al. (2009), Thorac Cardiovasc Surg, Turkey. Case report	14 yr male, SW in right 6th icr paravertebrally, stable with knife in place	Right posterolat thoracotomy (knife in situ), at removal bleeding from atrio- inferiocaval junction	Repair on CPB, discharged on 7th postop day	
[36] Gwely et al. (2010), Thorac Cardiovasc Surg, Egypt. Retrospective study	73 pts operated for cardiac SW (1998–2008)	Unstable 35%, 20% cardiac arrest prior to EDT. Mortality 23%	Poor prognosis: cardiopulmonary resuscitation (mortality rate 68%), EDT (67%) and shock (50%) on admission	Dead on arrival excluded

AVR - aortic valve replacement, CABG - coronary artery bypass, CPB - cardiopulmonary bypass, CX - circumflex artery, ED - emergency department, EDT - emergency department thoracotomy, FAST - focused assessment with sonography in trauma, GCS - Glasgow coma scale, GSW - gunshot wound, LA - left atrium, LAD - left anterior descendent artery, LV - left ventricle, OPCAB - off pump coronary artery bypass, OR - operating room, PDA - posterior descendent artery, RA - right atrium, ROSC - return of spontaneous circulation, RV - right ventricle, SVG - saphenous vein graft, TEE - transesophaegeal echocardiography, VF – ventricular fibrillation.

## Case presentation

A 28-year-old male was admitted to the emergency department (ED) with a 5 cm stab wound (SW) under his left nipple. Pre-hospital treatment included insertion of a left chest drain due to dyspnoea, but this was clamped during transport because of massive hemorrhage. On admission, he was self-ventilating, with palpable carotid pulses, but without a measurable blood pressure. He was agitated and pale with a Glasgow coma score of 12 since he could open his eyes, localize pain and speak. The blood pressure ranged from 80/60 to 100/60 mmHg after starting intravenous fluid therapy and he had a tachycardia of 100–120 beats per minute. When the clamp was removed from the chest drain, 650 ml of blood was rapidly drained. The chest x-ray showed persisting hemothorax and atelectasis and an additional drain was inserted. The arterial saturation varied from 86% to 98% and blood gas analysis showed a haemoglobin of 12.6 g/l, pH 7.17, base excess −9 and lactate 5.5 mmol/l. Focused Assessment with Sonography in Trauma (FAST) revealed no blood in the pericardium and upper abdomen. The neck veins were not distended and so the patient received transfusion of 1500 ml of crystalloid fluid and 250 ml of red cells. The blood pressure decreased as soon as the intravenous therapy was reduced, the tachycardia did not resolve and the patient was therefore transferred to the operating room.

**Figure 1:**
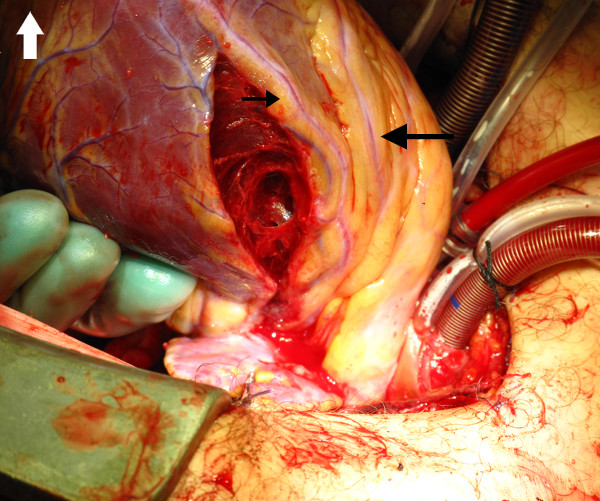
**The left ventricular injury almost penetrating the left ventricular wall, notice the left anterior descending coronary artery (large black arrow) with the first diagonal branch (small black arrow).** All the photos are taken from the anaesthesiologist point of view and the white arrow indicates the caudal direction.

**Figure 2:**
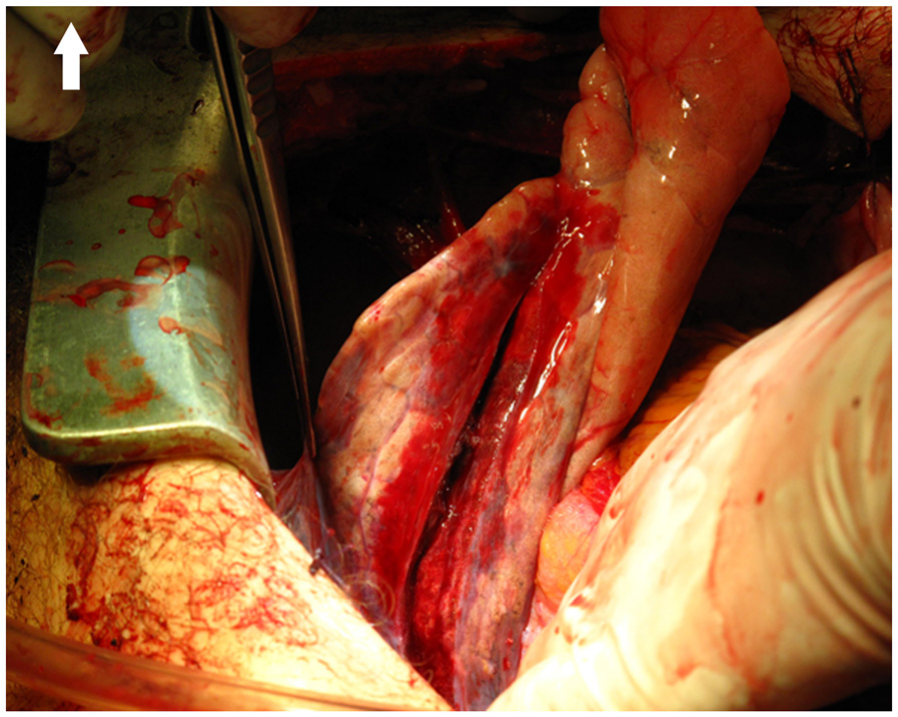
The injured left lung (upper lobe, lingula).

**Figure 3:**
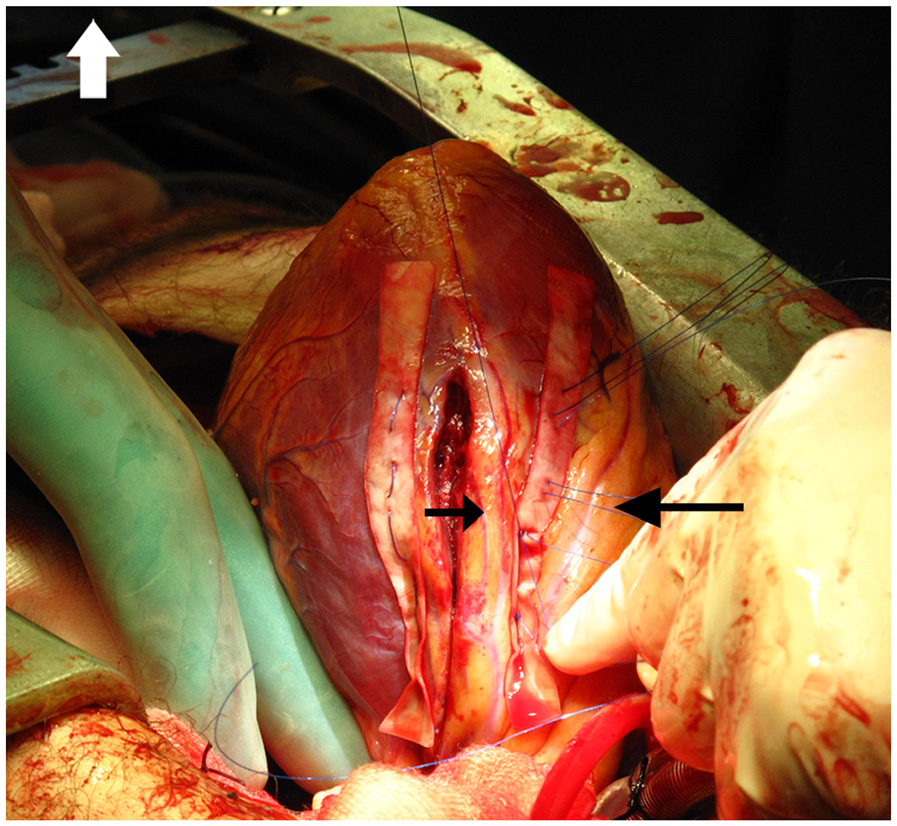
The wound repair with bovine pericardial strips.

**Figure 4:**
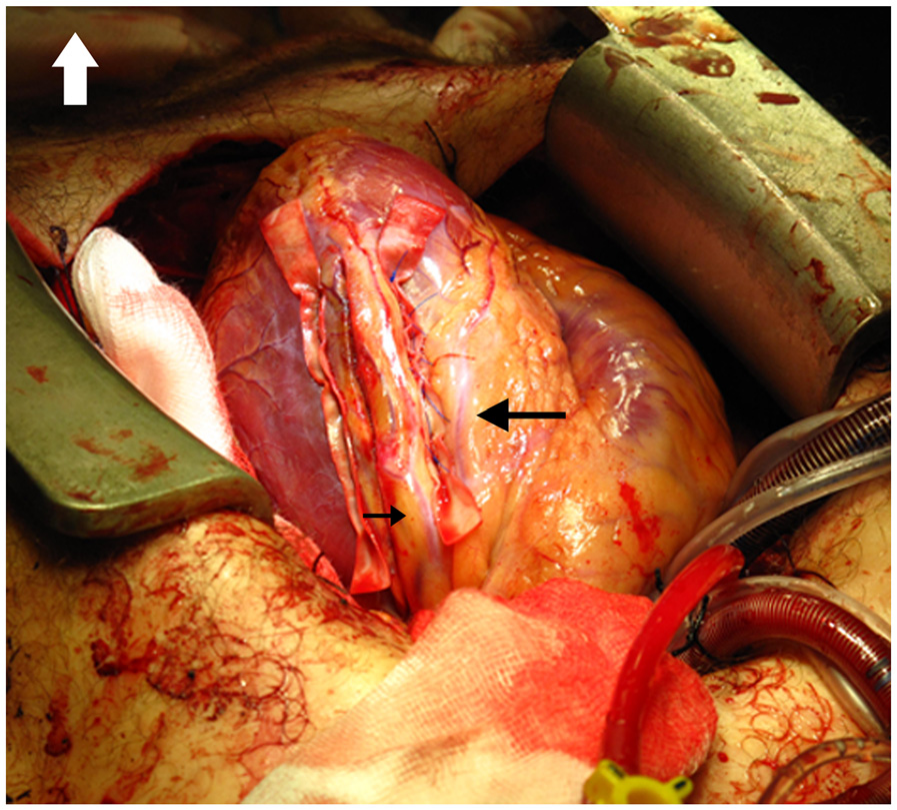
The completed repair of the left ventricular wound.

After intubation, the ECG showed ST elevation and a median sternotomy incision was rapidly performed. The pericardium was opened and although there was a clot ventral to the heart, there were no signs of cardiac tamponade. There was a 6 cm cut in the lateral pericardium corresponding to the stab wound in the chest and a 7 cm, almost transmural wound in the left ventricle, parallel to a major diagonal branch (Figure 1). The wound was not bleeding. A 5 cm stab wound in the left lung (Figure 2) was sutured and cardiopulmonary bypass (CPB) was established. The cardiac injury ended close to the origin of the left main stem and crossed the left atrium. The ventricular wound was repaired with single mattress sutures reinforced by strips of bovine pericardium (Figures 3, 4) without arresting the heart and without cross-clamping the aorta. The left atrium was sutured using 5/0 Prolene (Ethicon). Blood appeared in the tracheal tube and bronchoscopy revealed ongoing bleeding from the left lung which required resection of the lingula. Weaning from CPB was initially unsuccessful and we suspected that there had been injury to the left main stem either caused by the initial stab or by the hemostatic sutures. The left anterior descending artery was grafted using the internal mammary artery and a vein graft was anastomosed to the circumflex artery. The patient was thereafter successfully weaned from CPB.

Post-operatively, the patient had signs of a stroke and a CT scan revealed a cerebral infarction. One week after surgery he was transferred to the neurological intensive care unit. After three weeks he was awake and self-ventilating. He was moved to his local hospital and was discharged after 6 weeks with only a minor deficit affecting the left upper extremity.

## Discussion

We report the case of a young male patient with a major cardiac stab wound combined with lung injury. Our patient was stabbed during a violent quarrel, thus being a typical stab victim, however, in Japan suicide attempts seem to be equally frequent [18,23]. In large series, gunshot wounds (GSW) are the predominant cause of cardiac penetrating trauma [2,4,6,29]. In Norway, this type of injury is obviously less common but still existing [37-39]. Knife is the most common weapon for stab injuries, followed by other sharp items such as screwdrivers [34], ice picks [19], chopsticks, pneumatic nailgun nails [14,20,40] but also curiosities as barb from a sting ray [28]. Fractured ribs or sternum are also reported to cause cardiac penetration [41]. Pneumatic nails might be shot without the patient noticing and cause surprise when detected by CT scan or eccocardiography imbedded in the heart [14,20]. The iatrogenic penetrations of the heart due to different medical devices (pacemaker leads, intracoronary stents, Amplatzer devices) are not discussed in this paper.

Penetrating cardiac wounds are mostly fatal either due to cardiac tamponade, exsanguination or coronary artery injury [1]. Clarke reports that of 1064 patients with stab wounds to the chest 104 were operated and 76 were found to have a cardiac injury [3]. The overall mortality was 10% giving an impression of low mortality in this particular group of cardiac injuries. However, when the data was put together with the mortuary report for the same time, the mortality for penetrating cardiac stab wounds was found to be 30%. Most of the studies are retrospective and the patient selection is determined by the survivors arriving at the hospital and ignorance of the mortuary data. Topal et al. report a mortality rate of 15% in 61 penetrating cardiac cases with predominantly stab wounds but state that “patients pronounced dead on arrival were not assessed in this study” [33]. The only known prospective study reports another reality with a mortality rate of 97% when multichamber penetrating injury is present [2]. Also Molina et al. reports high mortality (67%) in a cohort with mainly stab wounds throughout the last decennium [4].

Our patient maintained suboptimal circulation for approximately two hours before undergoing surgery. The time span taken into consideration, our patient was extremely lucky as the outcome is usually poor when the time from trauma to surgery increases [5,6]. An Israeli study of 14 patients reports 100% survival (9 SW, 2 GSW, 1 shrapnel injury and 1 multi trauma) with the mean time from injury to surgery of 37 min [7]. In addition to fast admission to surgery, this outstanding result may also be due to the fact that all patients had single chamber injuries and no coronary artery injury. According to Burack et al., patients with penetrating mediastinal trauma triage themselves between operative intervention or evaluation and observation as they present either stable or unstable on admission. In this retrospective study the authors present 207 patients of which 72 were unstable [10]. Of these 15% had cardiac injury with 18% survival when explored in the ED. The survival rate was 71% when patients with penetrating cardiac injury reached the operating room. All patients having cardiac injury in this study were unstable (authors criteria: traumatic cardiac arrest or near arrest and an emergency department thoracotomy (EDT); cardiac tamponade; ATLS grad III shock despite fluid resuscitation; chest tube output >1500 ml at insertion; chest tube output >500 ml in the initial hour; massive hemothorax after chest tube input). The study does not report the use of CPB.

In our patient, there was a large stab wound of the left ventricle running parallel to the diagonal artery as well as a stab wound in the left atrium. Regarding the location of penetrating cardiac injury, the right ventricle is the most common due to its ventral anatomical position, followed by the left ventricle, right atrium and left atrium [2,3,11]. The patients with a single right ventricle injury are mostly salvagable whereas those with multichamber injuries have a very high mortality [2,4,21]. The concomitant injury of the lung in our patient is not a rarity [3]. Our patient did not suffer from cardiac tamponade as there was a large opening to the left pleural cavity through the wound in the pericardium. This probably saved his life, although profound hypovolemia can conceal signs of cardiac tamponade leading to delayed diagnosis [36]. However, cardiac tamponade in the reviewed studies is not a prognostic factor regarding survival [2,33].

The role of CPB has been debated in trauma surgery, espescially when it comes to penetrating cardiac wounds [6,21]. Some series present large cohorts of penetrating cardiac injury without use of CPB [3-5]. In case of complex cardiac injuries with multichamber lacerations the advantages of a bloodless and still operating field is obvious [6,20,21]. The required heparinisation for CPB might be deleterious in a trauma patient. However, if the bleeding source or sources can be controlled, the risk of further profound haemmorhage is low. On the other hand, full heparisation might cause severe morbidity, and CPB might initiate consumptive coagulopathy and profound systemic inflammatory reaction [28]. Off pump cardiopulmonary bypass is an alternative when it comes to coronary artery lesions [16,22,25]. Establishing CPB in arrested patients or patients in deep haemorrhagic shock is not favourable for the outcome [6]. It could be debated whether or not the aorta should have been cross-clamped in our patient during repair of the left ventricular wall and coronary bypass surgery, but the ECG changes and the suspicion of pre-existing ischemia due to sustained pre-operative hypoperfusion, persuaded us to leave the aorta unclamped in this particular case.

Peroperative fluorescent angiography is a reliable tool to identify suspect coronary artery involvement peroperatively either caused by the injury itself or the surgical procedure [15], unfortunately this technique was not available at our OR. Cardiac stabbings might lead to initially unidentified additional injuries which become apparent first several weeks to years later [8,18]. One study with a large series of patients report that these injuries seldom need surgical treatment [5]. There is consensus that echocardiographic assessment should be provided during the hospital stay [5,11].

On admission to the ED, our patient was given a high Glasgow coma score (GCS), yet post-operatively was found to have had a cerebral injury. Unfortunately, the patient was foreign, and despite speaking, nobody could assess his verbal response adequately. Furthermore, he received an intravenous injection of Ketalar a few minutes after admission, following which he needed assisted manual ventilation and was no longer able to communicate. The initial GCS was later reconsidered and probably the patient suffered from major hypoxia in the pre-hospital phase. Nevertheless the patients with lower GCS have poor outcome, Asensio still reports a high mortality rate (27%) for patients with Glasgow Coma Scale >8 [2]. However, in an emergency room thoracotomy material GCS was found to be a predictor of survival, despite none of the patients had a score >7 [29]. In our patient, it is possible that CPB might have caused cerebral injury by embolization or by giving an insufficient cerebral perfusion pressure. With pre-existing cerebral damage, the standard perfusion pressure during CPB in our patient (mean arterial pressure 50–60mmHg) might not have been high enough to meet the needs of the brain already damaged by hypoperfusion.

Patients with a simple penetrating cardiac injury might be successfully managed without a cardiac surgeon present [2,3]. However, repair of a severe wound of the left ventricle and the complications that can arise will require the surgical skills of a cardiac surgeon, as demonstrated in the present study and the likelihood of survival will be considerably increased by the immediate availability of a cardiac surgical service. The cases where initial tamponade was managed at a lower trauma care center with further transfer for definite surgery, witness of general surgeon`s competence of the initial management of these patients [13,28]. In our level I trauma center, a cardiothoracic surgeon in the trauma team has been practiced for decades and we believe provides optimal management of patients with penetrating cardiac trauma.

## Conclusions

We present a complicated case of a young male patient with a chest stab wound who served the trauma team both diagnostic and treatment challenges. We provide the reader a review of literature of the last 15 years publications on penetrating cardiac injury, focusing on stab wounds. Our patient suffered a stroke which origin could be multigenetic, prehospital hypoperfusion, air emboli due to major lung injury and/or insufficient perfusion pressure or microemboli during the cardiopulmonary bypass. The patient in our study survived with minor sequelae due to coordinated work of the trauma team in charge. In conclusion, if the patient with a penetrating stab wound in the heart is not obviously dead on arrival, an attempt for cardiac repair should be done with or without CPB.

## Competing interests

The authors declare that they have no competing interests.

## Authors’ contribution

Both authors were operating surgeons regarding the presented patient case. TT provided the idea of the article. M-L K drafted the initial manuscript while both authors worked on improvement and refining of the final manuscript. Both authors read and approved the final manuscript.
